# Synergistic Valorization of *Hevea brasiliensis*-Derived Spent Mushroom Substrate and *Elaeis guineensis* Fibers for Energy-Efficient Biocomposite Thermal Insulation Panels

**DOI:** 10.3390/biomimetics11050329

**Published:** 2026-05-08

**Authors:** Mohammad Aliff Shakir, Junfeng Zhu, Abdul Khalil H.P.S., Mardiana Idayu Ahmad

**Affiliations:** 1 School of Industrial Technology, Universiti Sains Malaysia, Gelugor 11800, Malaysia; maliffshakir@usm.my (M.A.S.); yqljzhujunfeng@student.usm.my (J.Z.); 2 Faculty of Business, Hospitality and Technology, Universiti Islam Melaka, Batu 28, Kuala Sungai Baru, Melaka 78200, Malaysia; akhalilhps@gmail.com; 3 Renewable Biomass Transformation Cluster, School of Industrial Technology, Universiti Sains Malaysia, Gelugor 11800, Malaysia

**Keywords:** biocomposite, natural waste fiber, sustainable, thermal insulation, building applications

## Abstract

Nature-inspired material design has gained increasing attention in the development of sustainable biocomposites for applications requiring the integration of structural performance and functional efficiency. However, many lignocellulosic composites still depend on synthetic binders and fail to achieve a strong effective interaction between constituents, resulting in suboptimal mechanical integrity and thermal behavior while limiting their environmental advantages. This study aims to develop binderless biocomposite panels from *Hevea brasiliensis*-derived spent mushroom substrate (SMS) and *Elaeis guineensis* empty fruit bunch (EFB) fibers, emphasizing the synergistic interaction between components for energy-efficient building applications. Chemical characterization revealed complementary roles, with EFB contributing a high cellulose content (57.60%) for reinforcement and SMS providing a higher lignin content (30.51%) for enhanced rigidity and natural binding. Panels were fabricated via hot pressing at a target density of 0.8 g/cm^3^ without additives. Mechanical properties were evaluated through specific flexural, tensile, internal bond, and impact testing, while thermal conductivity and thickness swelling were used to assess functional performance. The 60% SMS with 40% EFB composition exhibited optimal performance, achieving a specific flexural strength of 20.26 MPa, a flexural modulus of 1943.76 MPa, tensile strength of 6.12 MPa, an internal bond strength of 2.06 MPa, an impact strength of 15.35 kJ/m^2^, a thickness swelling of 44.80%, and a thermal conductivity of 0.234 W/m.K. These results demonstrate that the combined effect of SMS and EFB in binderless biocomposites derived from secondary products offers a promising biomimetic pathway for designing recyclable, high-performance materials suitable for sustainable and energy-efficient construction systems.

## 1. Introduction

The increasing demand for sustainable materials driven by resource depletion and environmental constraints has intensified the search for renewable alternatives to petroleum-based products in manufacturing industries. Among these, lignocellulosic secondary products have gained significant attention as potential substitutes for synthetic materials due to their renewability, biodegradability and favorable mechanical characteristics [1]. Common by-products such as spent mushroom substrate [2], oil palm residues [3], banana trunk fibers [4], coconut husk [5] and sugarcane bagasse [6] are generated in substantial quantities from agro-industrial activities [7]. Despite their high availability and low economic value, a large proportion of these biomass resources remain underexploited. In addition to their cost-effectiveness, natural waste fibers offer structural integrity and environmental compatibility, making them suitable for engineering applications. However, inadequate management and disposal of secondary products may contribute to environmental burdens, thereby highlighting the importance of systematic waste valorization into high-value functional materials [8].

Among secondary products, spent mushroom substrate (SMS) constitutes a major by-product of the mushroom cultivation industry and represents a largely underutilized lignocellulosic resource. Initially employed as a growth medium, SMS undergoes biological degradation during fungal cultivation, resulting in a residual biomass enriched with partially decomposed cellulose, hemicellulose and lignin [9]. In Malaysia and other mushroom-producing regions, rubberwood sawdust is widely used as the cultivation substrate due to its favorable lignocellulosic composition, which consequently influences the chemical profile of the resulting SMS [10,11]. Following harvesting, substantial quantities of SMS are generated, composed primarily of residual mycelium and structurally modified plant fibers. Previous studies report that SMS contains approximately 38–46.6% cellulose, 25–34.5% lignin, and 19–27.7% hemicellulose with the remaining fraction consisting of minor organic constituents [12]. Recent studies report that mushroom cultivation generates approximately 85 million tonnes of spent mushroom substrate (SMS) annually worldwide, underscoring the escalating scale of biomass waste associated with modern mushroom production [13]. Notably, the production of 1 kg of oyster mushrooms generates approximately 5 kg of residual substrate, underscoring the scale of biomass disposal and the urgency of developing value-added applications [14].

The oil palm industry represents one of the largest agro-industrial sectors in Southeast Asia and generates substantial quantities of lignocellulosic residues during palm oil extraction. Although fresh fruit bunches (FFB) are processed efficiently, only approximately 21–24% of the biomass is converted into palm oil [15,16]. The remaining fraction is discarded as by-products, including palm kernel shells, mesocarp fibers, and empty fruit bunch (EFB) fibers. Among these residues, EFB fibers are produced in particularly large volumes and have emerged as promising raw materials for value-added applications. Chemically comparable to hardwood fibers, EFB contains a high cellulose content ranging from 43% to 65%, which contributes significantly to its tensile strength and reinforcement capability in composite systems [17]. The high availability and favorable structural composition of EFB position it as a viable alternative to conventional cellulose-based reinforcement materials in sustainable panel production [18].

The valorization of SMS and EFB fibers into engineered composite panels represents a promising pathway for converting secondary products into high-performance functional materials. In such systems, multiple constituents are strategically integrated to achieve enhanced structural and thermal performance relative to their individual components. The performance of bio-based composite panels is strongly governed by fiber morphology, interaction bonding mechanisms, density distribution and processing parameters during fabrication [19]. Recent studies have explored the incorporation of natural waste fibers into insulation-oriented panels, emphasizing their low density, renewable origin and cost-effectiveness [20]. In addition to environmental benefits, these lignocellulosic composites exhibit favorable mechanical characteristics and inherent thermal resistance derived from their cellular microstructure. Nevertheless, achieving an optimal balance between mechanical integrity and thermal insulation performance remains a critical challenge, especially in binderless or low-adhesive systems.

The mechanical performance of composite panels is strongly influenced by the incorporation of reinforcement fibers and the strategic control of fiber percentage composition during fabrication. Fiber attributes including length, aspect ratio, diameter and surface morphology will govern stress transfer efficiency, inter-fiber entanglement and load distribution within the composite network [21]. In layered or hybrid systems, the relative proportion of each fiber type critically determines density gradients, compaction behavior, and interfacial compatibility between core and face layers. An excessive proportion of reinforcement fibers may disrupt structural homogeneity, increase void formation and weaken inter-particle bonding, ultimately reducing mechanical integrity. Conversely, insufficient reinforcement limits stress transfer capability [22]. However, existing studies on binderless lignocellulosic composites have predominantly focused on widely available fibers such as empty fruit bunch (EFB), while the utilization of spent mushroom substrate (SMS) as a functional component remains limited. In addition, most studies employ homogeneous mixing approaches, with limited exploration of layered or sandwich-structured configurations that could better exploit the distinct morphological and compositional characteristics of different fibers. Furthermore, the influence of fiber percentage composition in hybrid SMS–EFB systems, in relation to thermal insulation performance, has not been systematically investigated. Therefore, a systematic investigation of fiber percentage composition in hybrid SMS–EFB systems is essential to achieve a balanced synergy between chemical composition, mechanical properties, physical properties and thermal insulation performance in binderless composite panels.

To address these limitations, this study proposes a binderless biocomposite architecture that strategically integrates spent mushroom substrate (SMS) as the core layer and empty fruit bunch (EFB) fibers as reinforcement-rich face layers. The design exploits the complementary lignocellulosic composition and morphological characteristics of both fibers, where SMS with higher lignin content and finer particle size, contributes to core densification and reduced moisture sensitivity. Meanwhile, EFB fibers characterized by higher cellulose content and elongated structure, enhance load-bearing capacity and stress transfer efficiency. Unlike conventional biocomposites that rely on chemical binders or single-fiber systems, the present work employs an untreated layered configuration to promote environmentally benign processing while engineering structural compatibility through controlled fiber percentage composition [23]. The influence of varying SMS and EFB fiber ratios on flexural strength, tensile strength, internal bond strength, impact resistance, thickness swelling, and thermal conductivity is systematically evaluated. Furthermore, the developed panels are benchmarked against commercial gypsum boards to assess their feasibility as sustainable insulation materials for energy-efficient building applications. This study establishes a structure–composition–property relationship framework for optimizing binderless biocomposite panels derived entirely from secondary products.

## 2. Materials and Methods

### 2.1. Raw Materials Preparation

**Figure 1:**
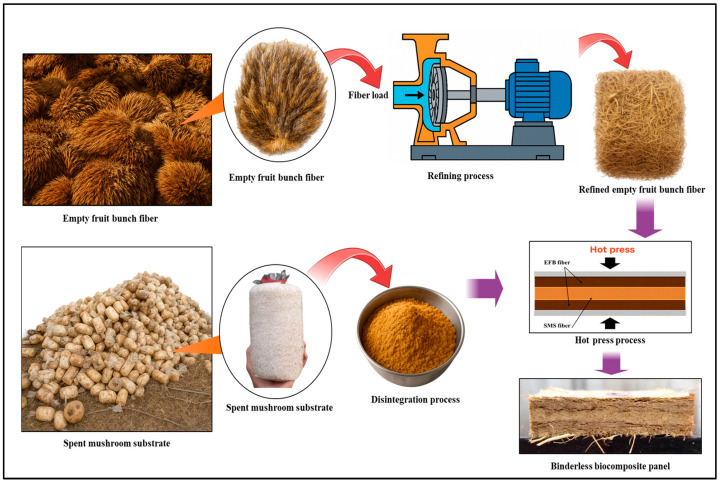
Preparation of SMS and EFB fibers and fabrication of the binderless biocomposite panel.

Spent mushroom substrate (SMS) fibers with an average fiber length of 650–700 µm were sourced from a mushroom cultivation facility in Tasek Gelugor, Penang, Malaysia. Empty fruit bunch (EFB) fibers derived from oil palm biomass were obtained from a local palm oil processing plant in Nibong Tebal, Penang, Malaysia. Prior to panel fabrication, EFB fibers were mechanically refined using a Waldron disk refiner (Andritz Sprout-Bauer Inc., Springfield, OH, USA) operated at a disk gap of 1.75 mm with a tolerance of ±0.25 mm as shown in Figure 1. The refining process produced fibers with an average length of approximately 60.000 µm. SMS fibers were manually extracted from post-harvest cultivation blocks and uniformly disintegrated to achieve a consistent particle distribution.

Biocomposite panels were fabricated at a target density of 0.8 g/cm^3^ using a sandwich-structured configuration, in which SMS fibers were designated as the core layer and EFB fibers were arranged as reinforcement-rich face layers as shown in Figure 1. EFB fiber mats were pre-formed to ensure a controlled thickness and homogeneous distribution before assembly. The assembled mats were then subjected to hot pressing at a temperature of 140 °C, under an applied pressure of 5 MPa for a duration of 40 min using a hot press machine (GOTECH Testing Machines Inc., Taichung, Taiwan) to achieve the target density of 0.8 g/cm^3^. To systematically investigate the influence of fiber percentage composition, panels were produced across a complete compositional gradient ranging from fully SMS-based biocomposites (100% SMS and 0% EFB) to fully EFB-based biocomposites (0% SMS and 100% EFB), with intermediate formulations of 80% SMS and 20% EFB, 60% SMS and 40% EFB and 40% SMS and 60% EFB. This design enabled a controlled evaluation of the transition from fine-particle-dominated core densification to long-fiber reinforcement effects within the sandwich-structured biocomposite system.

### 2.2. Chemical Composition Analysis

The chemical composition of SMS fiber and EFB fibers was determined in accordance with standardized ASTM procedures for lignocellulosic materials. Prior to analysis, fiber samples were oven-dried to a constant mass and ground. Extractive content was quantified using Soxhlet extraction following ASTM standard D1107-96 [24]. Acid-insoluble lignin content was determined in accordance with ASTM D1106-96 through sulfuric acid hydrolysis and subsequent gravimetric quantification of the insoluble residue [25]. Alpha-cellulose content was measured using ASTM D1103-60, involving alkaline extraction to remove hemicellulose fractions [26]. All chemical analyses were conducted in triplicate and average values were presented. This compositional characterization enabled the correlation of cellulose, hemicellulose, and lignin fractions with the mechanical and thermal behavior of the fabricated biocomposite panels.

### 2.3. Material Characterization and Testing

#### 2.3.1. Mechanical Properties

The flexural properties of the biocomposite panels were evaluated using a three-point bending test in accordance with JIS A 5908 (2003) [27]. Specimens were prepared with standardized dimensions as specified by the standard and testing was conducted using an Instron Model 5582 Universal Testing Machine (Instron, Norwood, MA, USA) with a speed accuracy of ±0.5%. The span length and crosshead speed were set according to the prescribed protocol to ensure a consistent stress distribution during bending [28]. Flexural strength and flexural modulus were calculated from the load-deflection curves obtained during testing. Specific flexural strength was determined by normalizing the flexural strength with respect to panel density to enable a performance comparison across different fiber percentage compositions.

Tensile strength was measured in accordance with ASTM D3039 (2000) [29]. Rectangular specimens were prepared and tested using a universal testing machine under monotonic loading until failure. The crosshead speed was maintained in accordance with the standard to ensure a uniform strain application. Tensile strength values were calculated from the maximum load sustained prior to fracture divided by the original cross-sectional area of the specimen. Care was taken to align specimens properly to prevent eccentric loading effects that could influence stress distribution.

Internal bond strength was determined following JIS A 5908 (2003) [27] to evaluate the bonding integrity within the panel structure. Specimens were cut to the required dimensions and bonded to metal loading blocks using a high-strength epoxy adhesive with fast curing characteristics to ensure a rigid attachment during testing. The adhesive was selected to provide sufficient bonding strength without influencing the failure behavior of the biocomposite specimens. The test was conducted under perpendicular tensile loading until failure occurred within the core region. Internal bond strength was calculated by dividing the maximum load by the bonded cross-sectional area, providing an indication of core compaction efficiency and inter-particle bonding within the binderless system.

Impact strength was evaluated using ASTM D256 (2006) to assess the resistance of the panels to sudden applied loads [30]. Specimens were prepared according to the specified geometry and tested using a pendulum-type impact tester. The absorbed impact energy was recorded and normalized by the cross-sectional area to obtain impact strength values expressed in kJ/m^2^. This test provided insight into crack initiation resistance and energy dissipation capability associated with varying fiber percentage compositions. All mechanical property measurements were conducted in triplicate for each fiber percentage composition and average values were reported.

#### 2.3.2. Physical Properties

The dimensional stability of the biocomposite panels was evaluated through thickness swelling measurements in accordance with JIS A 5908 (2003) [27]. Specimens were cut to the prescribed dimensions, and their initial thickness was measured at multiple locations using a digital micrometer with an accuracy of ±0.01 mm to ensure measurement precision. All physical property measurements were conducted in triplicate for each fiber percentage composition and average values were reported.

#### 2.3.3. Thermal Characterization

The thermal conductivity of the biocomposite panels fabricated was determined using the transient plane source (TPS) technique in accordance with ASTM C1045-07 [31]. Measurements were conducted with a Hot Disk Thermal Constants Analyzer (TPS 2500) (Hot Disk AB, Gothenburg, Sweden), which determines thermal transport properties based on the transient temperature response of a planar heat source positioned between two specimens. Each test was performed over a duration of 20 s with a controlled temperature increase of approximately 1.35 K to minimize moisture redistribution and microstructural alteration during heating. The composite samples were evaluated in triplicate to ensure reproducibility and the reported values represent the mean of three independent measurements.

## 3. Results

### 3.1. Lignocellulose Composition

**Figure 2:**
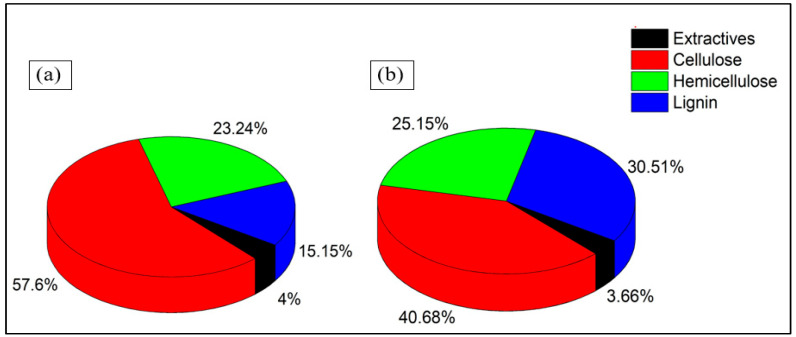
Lignocellulosic composition of natural fibers used for composite fabrication: (**a**) EFB fiber and (**b**) SMS fiber.

As presented in Figure 2, EFB fibers are characterized by a cellulose content of 57.60%, whereas SMS contains 40.68%. This substantial difference directly influences the mechanical reinforcement capability of the developed biocomposites. Cellulose possesses a semi-crystalline structure and functions as the principal load-bearing component in lignocellulosic materials [24,25]. Therefore, the higher cellulose fraction in EFB enhances tensile stiffness and improves stress transfer efficiency under flexural loading.

### 3.2. Specific Flexural Strength

**Figure 3:**
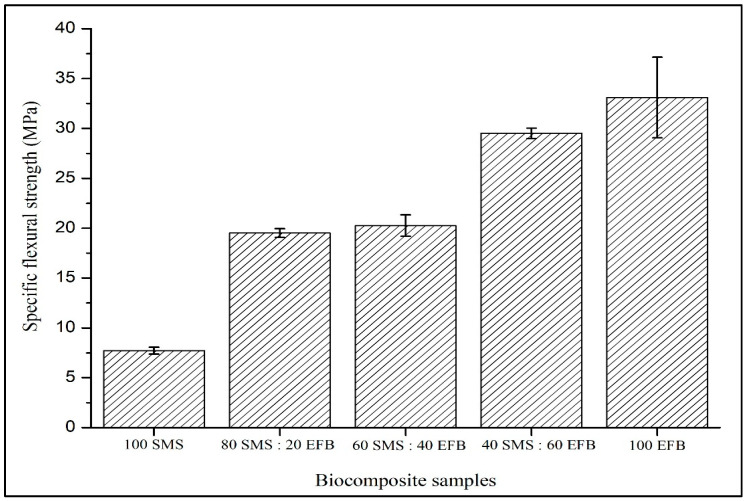
Effect of SMS and EFB fiber percentage composition on the specific flexural strength of the biocomposite samples.

Figure 3 shows that the specific flexural strength increased progressively with increasing EFB fiber percentage composition. The fully EFB-based configuration achieved the highest value of 33.1 MPa, whereas the fully SMS-based panel recorded the lowest value of 7.73 MPa. The hybrid panel containing 40% SMS and 60% EFB exhibited a relatively high strength of 29.53 MPa. In contrast, intermediate compositions of 80% SMS and 20% EFB and 60% SMS and 40% EFB demonstrated moderate values of 19.51 MPa and 20.26 MPa, respectively. These results clearly indicate that bending efficiency is strongly governed by fiber composition with performance improving as EFB content increases.

### 3.3. Flexural Modulus

**Figure 4:**
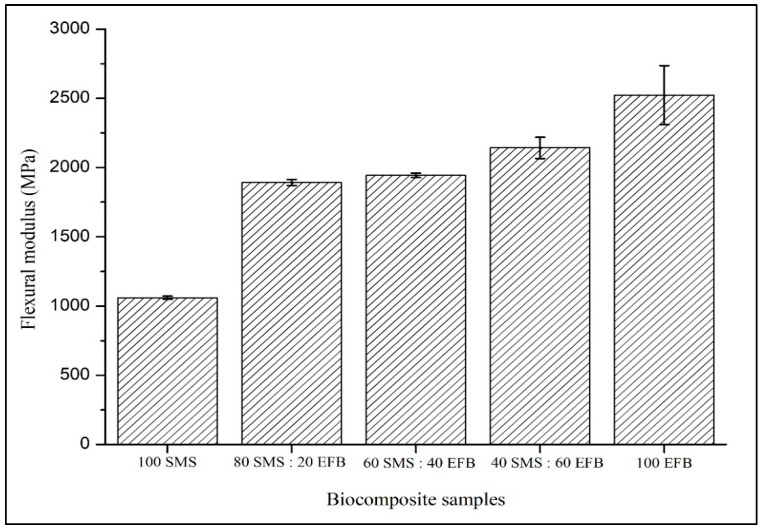
Effect of SMS and EFB fiber percentage composition on the flexural modulus of the biocomposite samples.

Figure 4 illustrates the influence of SMS and EFB fiber percentage composition on the flexural modulus of the biocomposite panels. The flexural modulus ranged from 1530.18 MPa to 2523.27 MPa. The lowest value of 1530.18 MPa was recorded for the panel containing 100% SMS, whereas the highest modulus of 2523.27 MPa was achieved at 100% EFB. The flexural modulus increased progressively with increasing EFB content, with the fully EFB-based panel exhibiting the highest stiffness. This trend confirms the dominant role of cellulose-rich EFB fibers in enhancing the elastic load transfer within the composite structure.

### 3.4. Tensile Strength

**Figure 5:**
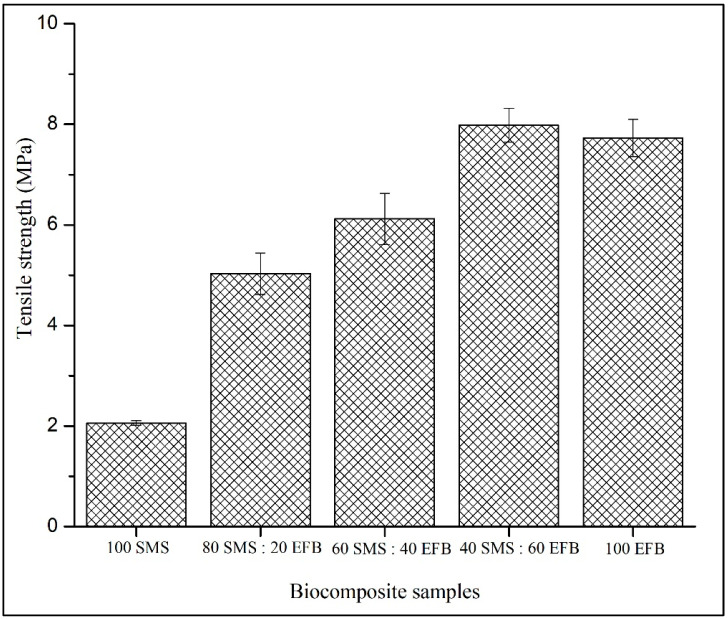
Effect of SMS and EFB fiber percentage composition on the tensile strength of the biocomposite samples.

Figure 5 illustrates the influence of SMS and EFB fiber percentage composition on the tensile strength of the biocomposite panels. The tensile strength ranged from 2.06 to 7.98 MPa. The lowest value of 2.06 MPa was recorded for the panel containing 100% SMS, whereas the highest tensile strength of 7.98 MPa was achieved at 80% EFB and 20% SMS. Although tensile strength generally increased with increasing EFB content, the configuration containing 40% SMS and 60% EFB exhibited a higher value than the fully EFB-based panel, indicating that optimal tensile performance occurs at an intermediate fiber percentage composition.

### 3.5. Internal Bond Strength

**Figure 6:**
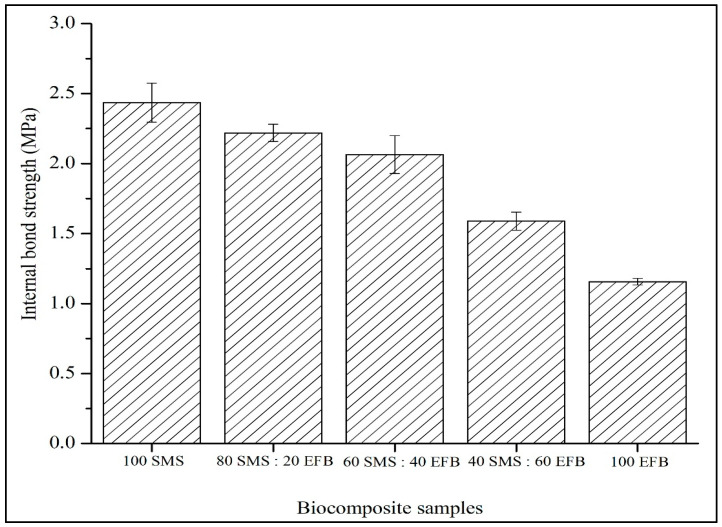
Effect of SMS and EFB fiber percentage composition on internal bond strength of the biocomposite samples.

Figure 6 illustrates the influence of SMS and EFB fiber percentage composition on the internal bond strength of the biocomposite panels. The internal bond strength ranged from 1.16 to 2.44 MPa. The lowest value of 1.16 MPa was recorded for the panel containing 100% EFB fiber, whereas the highest value of 2.44 MPa was achieved at 100% SMS fiber. Overall, internal bond strength decreased progressively as the proportion of EFB fibers increased. Furthermore, all hybrid configurations exhibited a higher internal bond strength than the fully EFB-based panel, demonstrating the contribution of SMS fibers to improved interaction within the composite structure.

### 3.6. Impact Strength

**Figure 7:**
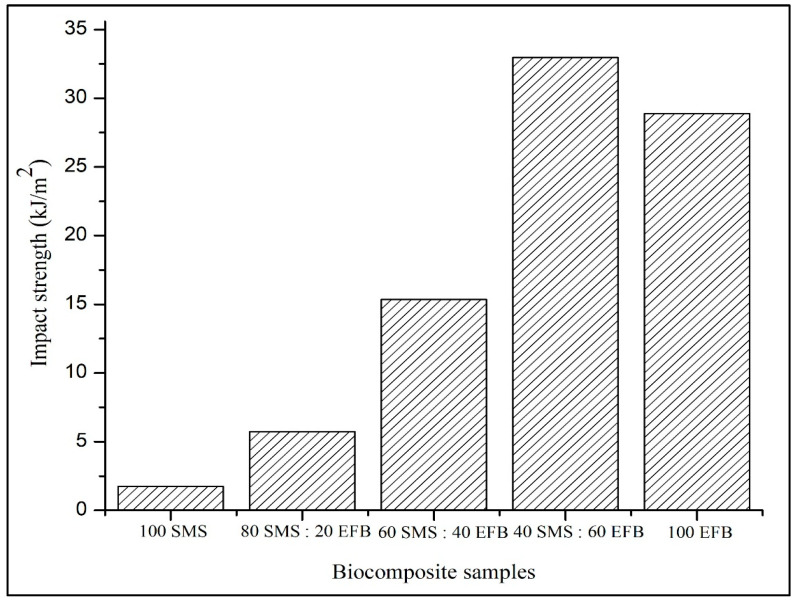
Effect of SMS and EFB fiber percentage composition on impact strength of the biocomposite samples.

Figure 7 presents the influence of SMS and EFB fiber percentage composition on the impact strength of the biocomposite panels. The impact strength ranged from 1.75 to 32.97 kJ/m^2^. The lowest value of 1.75 kJ/m^2^ was recorded for the panel containing 100% SMS fiber, whereas the highest value of 32.97 kJ/m^2^ was achieved at 40% SMS and 60% EFB. An overall increasing trend in impact resistance was observed with increasing EFB content. However, the fully EFB-based configuration did not exhibit the maximum value, indicating that optimal performance occurs at an intermediate composition.

### 3.7. Thickness Swelling

**Figure 8:**
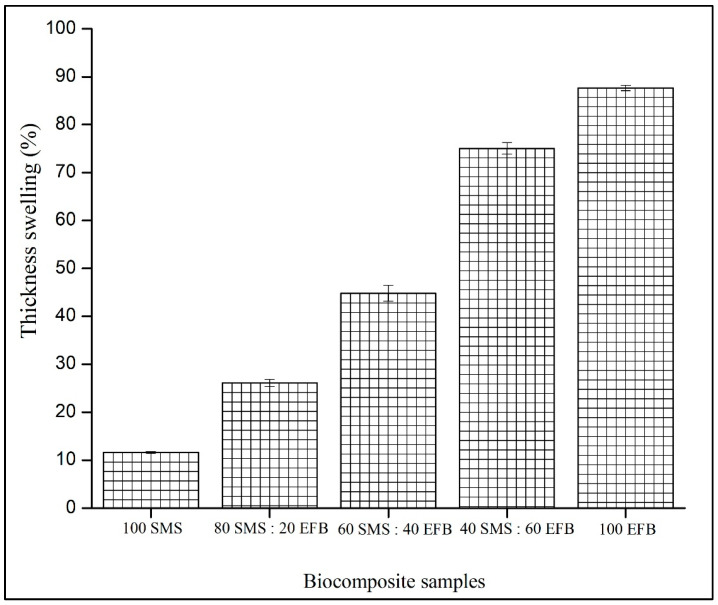
Effect of SMS and EFB fiber percentage composition on thickness swelling of the biocomposite samples.

Figure 8 illustrates the effect of SMS and EFB fiber percentage composition on the thickness swelling of the biocomposite panels. The thickness swelling ranged from 11.63% to 87.70%. The lowest value of 11.63% was recorded for the panel containing 100% SMS fiber, whereas the highest thickness swelling of 87.70% was observed at 100% EFB. Overall, thickness swelling increased with increasing EFB fiber percentage composition. Furthermore, all hybrid configurations exhibited a lower thickness swelling than the fully EFB-based panel, indicating improved dimensional stability when SMS fibers were incorporated.

### 3.8. Thermal Conductivity Performance

The biocomposite formulation containing 60% SMS and 40% EFB was selected for thermal conductivity evaluation based on its balanced mechanical performance and improved dimensional stability compared to other compositions. Although the configuration containing 40% SMS and 60% EFB exhibited superior tensile and flexural properties, the 60% SMS and 40% EFB panel demonstrated a lower thickness swelling while maintaining satisfactory structural integrity. This balance between mechanical strength and dimensional stability makes it the most suitable candidate for assessing thermal insulation performance. From a thermal perspective, this composition also provides a balanced internal structure. A higher proportion of SMS consisting of finer particles, tends to increase densification by filling void spaces, which may enhance solid-phase heat conduction [32]. In contrast, a higher proportion of EFB fibers introduces greater porosity due to their elongated morphology. While porosity is beneficial for thermal insulation, excessive or large, interconnected voids may facilitate convective heat transfer and reduce insulation efficiency. Therefore, the 60% SMS and 40% EFB composition represents a balance between densification and controlled pore distribution, which is critical for achieving a low thermal conductivity in lignocellulosic composites.

As presented in Table 1, the thermal conductivity of the selected biocomposite panel was 0.234 W/m.K. This value is considerably lower than reported thermal conductivity values for gypsum panels, which range between 0.314 and 0.390 W/m.K, indicating improved resistance to heat transfer compared to conventional gypsum-based materials [33,34,35]. However, the thermal conductivity remains higher than that of high-performance insulation materials such as polyurethane foam and mineral wool, which typically range between 0.020 and 0.046 W/m.K. In comparison, the developed biocomposite exhibits performance comparable to conventional wood-based panels such as MDF (Medium Density Fiberboard). These results suggest that the developed material is more suitable as a sustainable and binderless alternative for moderate insulation applications rather than as a direct replacement for high-performance synthetic insulation materials.

## 4. Discussion

### 4.1. Lignocellulose Composition

Conversely, as shown in Figure 2, SMS fibers exhibit a higher lignin content of 30.51% compared to 15.15% in EFB. The elevated lignin fraction modifies the functional role of SMS within the composite system. Due to its highly crosslinked aromatic structure, lignin contributes to rigidity and thermal stability while reducing polymer chain mobility. In binderless systems, lignin may also assist in inter-particle adhesion during hot pressing through thermoplastic softening behavior, thereby promoting core densification [42]. However, the lower polarity of lignin relative to cellulose can limit hydrogen bonding efficiency, which contributes to the reduction in tensile and flexural strength when SMS becomes the dominant phase.

In addition, lignin exhibits a relatively low polarity and a pronounced hydrophobic character due to its highly crosslinked aromatic structure [43]. This results in a heterogeneous, amorphous, and partially porous biopolymer network. Such structural characteristics influence the interfacial interactions within the composite system, where reduced polarity limits hydrogen bonding with hydrophilic cellulose, thereby affecting stress transfer efficiency and contributing to variations in mechanical performance. From a thermal perspective, the disordered structure of lignin disrupts phonon transport pathways, as phonon propagation is more efficient in ordered and crystalline domains such as cellulose [44]. Consequently, the hydrophobic and irregular nature of lignin enhances phonon scattering and reduces solid-phase thermal conductivity, which contributes to improved thermal insulation performance.

Hemicellulose contents are 25.15% in SMS fiber and 23.24% in EFB fiber, indicating relatively comparable levels of amorphous polysaccharides in both fibers. Owing to its hydrophilic nature, hemicellulose influences moisture uptake and dimensional stability [45]. However, the small difference between the two fibers suggests that variations in swelling behavior are more strongly governed by compaction characteristics and structural configuration than by the hemicellulose fraction alone. From a thermal insulation perspective, chemical composition contributes to solid-phase heat transfer behavior. Cellulose being more ordered and crystalline, facilitates a slightly higher phonon transport compared to lignin [46]. In contrast, the amorphous aromatic network of lignin restricts phonon mobility and exhibits a lower intrinsic thermal conductivity. Therefore, the higher lignin content in SMS fiber could contribute to reduced solid-phase thermal conduction within the core layer. Nevertheless, it is important to note that overall thermal conductivity remains predominantly governed by porosity and density, with chemical composition exerting a secondary but synergistic effect.

The hybridization of cellulose-dominant EFB (57.60% cellulose) and lignin-enriched SMS (30.51% lignin) thus enables the controlled tuning of mechanical reinforcement and thermal resistance. EFB enhances structural stiffness through its higher cellulose fraction and elongated morphology, whereas SMS promotes densification and thermal stability due to its chemical composition and finer particle structure. This compositional complementarity underpins the optimized balance between mechanical integrity and thermal insulation performance observed in the biocomposite panels fabricated using the sandwich method. It should be noted that the SMS fibers used in this study originate from a biologically treated lignocellulosic substrate, where enzymatic degradation during mushroom cultivation partially alters the original lignocellulosic structure. In particular, water-soluble components such as sugars may have been reduced prior to analysis, which could influence the measured chemical composition and its interpretation.

### 4.2. Specific Flexural Strength

Based on Figure 3, the specific flexural strength increases with increasing EFB content due to its higher cellulose fraction and superior reinforcement capability. EFB contains a higher cellulose fraction (57.60%) and lower lignin content (15.15%), which enhance tensile stiffness and load-bearing efficiency under bending. As cellulose serves as the primary structural polymer in lignocellulosic materials, its higher concentration promotes more effective stress transfer along the outer layers of the panel, where tensile stresses are maximized during flexural loading. In contrast, SMS fibers contain lower cellulose (40.68%) and a higher lignin content (30.51%). Although lignin contributes to rigidity and thermal stability, its lower polarity limits hydrogen bonding potential, thereby reducing reinforcement continuity when SMS becomes predominant [47].

From a structural mechanics perspective, flexural performance is governed by fiber continuity, the interaction between fibers and crack-bridging capability. The elongated and stranded morphology of EFB fibers promotes mechanical interlocking and enhances load redistribution across the panel thickness [48]. This observation is consistent with recent studies, which report that natural fibers with higher cellulose content and elongated morphology contribute to improved flexural performance through enhanced stress transfer and crack-bridging mechanisms [49]. Conversely, increasing SMS content introduces finer particles that may generate localized stress concentration sites and microvoids in binderless systems. In the absence of synthetic binders, structural integrity relies heavily on fiber entanglement and hydrogen bonding. Therefore, insufficient reinforcement continuity weakens the load-bearing network and reduces bending resistance [33].

Fracture behavior further supports this interpretation. Layered composite systems often exhibit sequential failure, where weaker core layers undergo initial shear deformation followed by fracture of stiffer face layers under increasing load [34,35]. However, the panels in this study predominantly displayed single-stage fracture behavior, suggesting effective interlayer compatibility between SMS and EFB fibers. In layered composite systems, failure typically occurs at the interface between layers when interfacial bonding is weak, resulting in non-simultaneous or delamination-type fracture. In such cases, interpretation based on macroscopic observation is necessary and the absence of detailed interfacial characterization may introduce uncertainty in identifying the exact failure mechanism. Therefore, in this work, the variation in flexural strength is primarily governed by fiber percentage composition rather than interfacial delamination. Collectively, these findings indicate that cellulose-rich EFB fibers function as the dominant reinforcement phase, whereas excessive SMS content disrupts crack-bridging efficiency despite contributing to core densification.

### 4.3. Flexural Modulus

Based on Figure 4, the increase in flexural modulus with increasing EFB content is consistent with the higher cellulose fraction (57.60%) and the elongated morphology of EFB fibers has enhance elastic load transfer under bending. Flexural modulus represents resistance to elastic deformation, so fiber continuity and the aspect ratio play a critical role in determining stiffness. The long-fiber architecture of EFB promotes network connectivity and improves stress redistribution across the outer layers of the panel, where bending stresses are concentrated [50]. The finer SMS particles may enhance core packing density and reduce internal void heterogeneity, thereby improving stress transmission between layers. However, SMS alone is insufficient to provide effective load transfer due to its shorter particle morphology and limited reinforcement continuity. The presence of long EFB fibers is essential to establish a continuous load-bearing network within the composite. This suggests that optimal stiffness is governed by a balance between reinforcement efficiency and internal compaction. The flexural modulus results indicate that stiffness in the biocomposite panels is controlled by a coupled morphological-compositional mechanism, where EFB fibers provide elastic reinforcement while SMS fibers contribute to structural densification within the composite configuration.

### 4.4. Tensile Strength

As shown in Figure 5, the increasing trend in tensile strength with higher EFB content is consistent with previous studies highlighting the superior reinforcement efficiency of EFB fibers due to their elongated and stranded morphology [51]. The long-fiber geometry promotes the formation of an interconnected fiber network within the composite, enabling efficient stress absorption and transfer in both longitudinal and transverse directions. Composites with enhanced reinforcement continuity are capable of sustaining higher tensile loads due to improved load redistribution mechanisms. This explains the overall increase in tensile strength as EFB content increases. The tensile strength obtained in this study (2.06–7.98 MPa) falls within the range reported in recent studies on binderless lignocellulosic composites, typically between 1.4 and 7.8 MPa, indicating that the developed biocomposite exhibits comparable mechanical performance despite the absence of synthetic binders [52]. However, the observation that the fully EFB-based panel did not exhibit the highest tensile strength suggests that long-fiber dominance alone does not guarantee optimal performance. Previous studies have reported that composites composed exclusively of long fibers are prone to fiber entanglement, resulting in inhomogeneous distribution, a limited intimate contact between fiber particles and non-uniform stress transfer [53,54]. Such structural irregularities may create localized stress concentrations that reduce effective tensile resistance.

The incorporation of SMS fibers at moderate proportions appears to mitigate these limitations. SMS fibers, characterized by a finer particle morphology, can infiltrate and occupy void spaces within the EFB fiber mat during hot pressing. This was qualitatively observed during fabrication, where SMS fine particles filled the inter-fiber gaps of the EFB network, promoting improved packing density and structural compatibility between layers. Similar hybrid fiber systems have demonstrated that fine particles enhance the interaction between components and stress distribution by reducing void heterogeneity [55]. The improved compatibility between SMS and EFB layers likely facilitated a more uniform stress uptake across the panel cross-section, leading to enhanced tensile strength. Therefore, it can be seen that the tensile performance in the developed biocomposite panels is governed not only by the geometrical structure of EFB fibers but also by the compatibility and synergistic interaction between SMS and EFB layers. The hybridization of two morphologically distinct fibers enhances structural homogeneity and improves stress transfer efficiency, resulting in superior tensile behavior compared to single-fiber systems.

### 4.5. Internal Bond Strength

In Figure 6, the internal bond strength reflects the tensile resistance perpendicular to the panel surface and is therefore highly sensitive to inter-particle adhesion and core densification. In binderless systems, bonding relies primarily on hydrogen bonding, mechanical interlocking and the thermoplastic softening of lignin during hot pressing [56]. SMS fibers contain a higher lignin content, which can contribute to improved bonding through partial softening and flow under pressing conditions. This may enhance contact between adjacent particles and increase cohesive strength within the core layer. When SMS becomes the dominant component, the internal bond strength increases due to enhanced core densification and improved inter-particle contact. The finer morphology of SMS particles promotes closer packing and reduces the internal void spaces within the panel core.

Conversely, increasing the EFB content results in a gradual reduction in the internal bond strength. The elongated and bulky structure of EFB fibers can reduce packing efficiency and increase void heterogeneity within the core region. Although EFB provides effective reinforcement under bending, its long-fiber morphology may limit intimate inter-particle contact under perpendicular tensile loading. Consequently in this work, internal bond performance is primarily governed by particle packing density and lignin-assisted adhesion rather than fiber length, explaining the superior bonding observed in SMS-rich configurations. It should also be noted that the failure of location during internal bond testing is an important consideration for layered composites. Ideally, failure should occur within the SMS-rich core region, indicating effective cohesion within the panel. However, if failure occurs at the SMS–EFB interface, the measured internal bond strength may reflect interlayer separation rather than true core cohesion. Therefore, careful observation of the failure mode is necessary to minimize interpretation errors in internal bond analysis. These findings demonstrate that internal bonding in the developed biocomposite panels is primarily governed by lignin-assisted adhesion and particle packing density. The superior bonding observed in SMS-rich configurations suggests the dominant role of fine particle morphology and higher lignin content in enhancing structural cohesion within the sandwich-structured biocomposite system.

### 4.6. Impact Strength

Impact strength in Figure 7 reflects the ability of the composite to absorb and dissipate energy under sudden loading conditions. The increasing trend with higher EFB content can be attributed to the elongated and stranded morphology of EFB fibers, which promotes the formation of a stronger fiber network within the composite structure [57]. The long-fiber geometry enhances crack-bridging, fiber pull-out and stress redistribution mechanisms, enabling the efficient absorption of impact energy. As reported in previous studies, composites with superior reinforcement continuity are capable of sustaining higher dynamic loads due to improved stress transfer efficiency [58]. The superior performance observed at 40% SMS and 60% EFB suggests a synergistic interaction between the two fiber types. While EFB provides the primary reinforcement, the presence of SMS fine particles appears to improve fiber distribution and structural homogeneity. During fabrication, SMS particles were observed to infiltrate the void spaces within the EFB fiber mat, enhancing packing density and improving the interaction between fiber layers. Similar hybrid systems have reported that fine particles reduce void heterogeneity and improve stress distribution across the composite thickness [55]. The improved compatibility between SMS and EFB fibers likely facilitated a more uniform energy absorption during impact loading.

In contrast, SMS-dominant configurations exhibited substantially lower impact resistance. As SMS fibers are characterized by a shorter particle morphology, they display comparatively brittle behavior under dynamic loading. The reduced fiber length limits the pull-out mechanisms and energy dissipation capacity, resulting in a more rapid crack propagation [59]. These findings indicate that impact performance is governed not solely by the reinforcement content but by the balance between fiber continuity and structural compactness. The results demonstrate that the hybridization of EFB fibers with SMS fibers enhances impact resistance through improved fiber network formation and the interaction between fiber components. Optimal dynamic performance is achieved at an intermediate fiber percentage composition rather than at a single-fiber dominance.

### 4.7. Thickness Swelling

In Figure 8, the increase in thickness swelling can be attributed to the release of compression stress accumulated during the hot pressing process. During fabrication, high pressure induces a substantial compression within the fiber network in binderless systems where structural integrity relies on hydrogen bonding between fiber particles. In the absence of synthetic binders, inter-fiber hydrogen bonds serve as the primary cohesive mechanism. Upon exposure to water, these hydrogen bonds are disrupted which results in the relaxation of previously stored compression stresses. This stress release phenomenon manifests as thickness expansion, commonly referred to as the spring-back effect, whereby the composite tends to revert toward its pre-pressed configuration [60,61]. The thickness swelling values obtained in this study (11.63–87.70%) are comparable to those reported in recent studies on binderless lignocellulosic composites, which typically range between [24.64–63.15%], indicating a similar dimensional stability behavior influenced by fiber hygroscopicity and structural characteristics [62]. This difference can be attributed to variations in particle size, where finer particles promote improved packing density and reduced void space, thereby limiting moisture-induced expansion.

The magnitude of this spring-back effect increased with the higher EFB fiber content. Morphologically, EFB fibers possess a low density and a bulky, elongated structure, requiring greater compression during panel formation to achieve the target density. The higher volume fraction of compressible EFB fibers leads to a greater stored elastic recovery during hot pressing. Consequently, when exposed to wet conditions, panels with a higher EFB percentage composition exhibit greater stress relaxation and thickness recovery. In contrast, SMS fibers, characterized by finer particle morphology, contribute to improved core packing and reduced elastic recovery.

Chemical composition also plays a critical role in moisture-induced swelling. EFB fibers contain higher cellulose and hemicellulose contents, both of which possess abundant hydroxyl groups that facilitate water absorption [63]. An increased water uptake promotes fiber expansion and enhances the spring-back effect. Conversely, SMS fibers have undergone partial lignocellulosic degradation during mushroom cultivation, resulting in a reduced hydrophilicity and a lower intrinsic swelling capacity [64]. This explains the lower thickness swelling observed at higher SMS compositions. In this work, the thickness swelling in the developed biocomposite panels is governed by a coupled mechanical-chemical mechanism involving compression stress release, hydrogen bond disruption, fiber compressibility and intrinsic hygroscopicity. Optimal dimensional stability is achieved at higher SMS percentage compositions due to enhanced core densification and reduced moisture sensitivity.

### 4.8. Thermal Conductivity Performance

In Table 1, the lower thermal conductivity of the biocomposite panel can be attributed primarily to its internal void structure. It has been widely reported that smaller and well-distributed void gaps reduce heat transfer due to the low thermal conductivity of entrapped air compared to the solid phases [65,66]. In the present study, the formation and distribution of voids were strongly influenced by the fine particle size of SMS fibers and the compressibility of EFB fibers during hot pressing. The SMS fine particles contributed to the refinement of internal pore structure, while the compressive behavior of EFB fibers facilitated controlled densification. Together, these effects disrupted continuous heat conduction pathways and reduced overall thermal conductivity. In contrast, the thermal conductivity of gypsum panels is strongly influenced by particle packing density, pore distribution and residual moisture content. The pore structure of gypsum-based materials is developed during calcination, hydration and subsequent drying processes, which govern internal air entrapment and solid-phase continuity [67]. Particle size also plays a critical role in determining thermal performance. Conventional gypsum panels commonly have excessively fine gypsum particles, which may lead to a denser packing, a reduced air void fraction and an increased solid-phase heat conduction [68,69]. Furthermore, retained moisture after fabrication can elevate thermal conductivity as water exhibits a significantly higher thermal conductivity than entrapped air [70]. Collectively, these factors contribute to the comparatively higher thermal conductivity observed in conventional gypsum panels.

Compared to conventional gypsum panels, the developed biocomposite demonstrates improved thermal insulation performance. Although its thermal conductivity remains higher than that of high-performance materials such as polyurethane foam and mineral wool, it is comparable to conventional wood-based panels such as MDF, indicating its suitability as a sustainable and binderless alternative for moderate insulation applications. In this work, the 60% SMS and 40% EFB biocomposite panel demonstrates improved thermal insulation performance compared to conventional gypsum materials. The combined effect of the SMS-induced pore refinement and the EFB structural compaction results in a structure that effectively limits heat transfer. These findings highlight the potential of the developed binderless biocomposite for sustainable building insulation applications.

## 5. Conclusions

This study successfully developed binderless biocomposite panels from *Hevea brasiliensis*-derived spent mushroom substrate and *Elaeis guineensis* empty fruit bunch fibers using a sandwich-structured configuration. The results demonstrate that the fiber percentage composition critically governs the mechanical-physical-thermal synergy of the developed system. Chemical composition analysis revealed that EFB contains 57.60% cellulose, which contributes to enhanced reinforcement efficiency. Meanwhile, SMS contains 30.51% lignin, contributing to core densification and a reduced moisture sensitivity. These compositional differences translated directly into performance variations across formulations. Mechanically, the 40% SMS and 60% EFB configuration achieved the highest tensile strength of 7.98 MPa and the highest impact strength of 32.97 kJ/m^2^, confirming the role of long-fiber reinforcement and hybrid fiber interaction. Meanwhile, the fully EFB-based panel reached a maximum specific flexural strength of 33.1 MPa and a flexural modulus of 2523.27 MPa. In contrast, the 100% SMS panel achieved the highest internal bond strength of 2.44 MPa, demonstrating superior core cohesion through lignin-assisted bonding and enhanced particle packing. It also exhibited the lowest thickness swelling of 11.63%, confirming a reduced spring-back behavior and improved dimensional stability. Thermally, the composite made of the 60% SMS and 40% EFB configuration achieved a low thermal conductivity of 0.234 W/m.K, representing a reduction of approximately 8 to 40% compared to reported gypsum panels (0.314–0.390 W/m.K). This result indicates that the developed biocomposite exhibits enhanced resistance to heat transfer, which is a critical requirement for thermal insulation materials used in building applications. The lower thermal conductivity can be attributed to the synergistic effect of a refined internal pore structure and a controlled densification, which disrupt the continuous heat conduction pathways within the material. Compared to conventional gypsum-based panels, the developed biocomposite demonstrates improved insulation performance. However, its thermal conductivity remains higher than that of high-performance insulation materials such as polyurethane foam and mineral wool, while being comparable to conventional wood-based panels such as MDF, indicating its suitability for moderate insulation applications. The results suggest a structure–composition–property relationship in the SMS–EFB binderless biocomposites. The hybridization strategy enables simultaneous optimization of mechanical integrity, dimensional stability and thermal insulation without synthetic binders. These findings confirm the potential of agricultural waste-derived biocomposite panels as sustainable thermal insulation materials for building applications. Future work will focus on further understanding and optimizing the developed biocomposite system. In particular, a detailed microstructural characterization will be conducted to better elucidate fiber interactions and internal structure. In addition, a thermal conductivity evaluation across a wider range of compositions will be performed to strengthen the structure–composition–property relationship.

## Figures and Tables

**Table 1:** Thermal conductivity comparison between the developed 60% SMS and 40% EFB biocomposite panel and conventional gypsum panels reported in previous studies.

Type of Panel	Thermal Conductivity (W/m.K)	References
Binderless biocomposite panel at (60% SMS and 40% EFB)	0.234	Present study
Gypsum panel	0.390	[36]
	0.314	[37]
	0.254–0.314	[38]
Polyurethane foam	0.031	[39]
Mineral wool	0.030–0.046	[40]
MDF panel	0.18	[41]

## Data Availability

The data presented in this study are available on request from the corresponding author.

## Abbreviations

The following abbreviations are used in this manuscript:

SMS	Spent mushroom substrate
EFB	Empty fruit bunch
TPS	Transient plane source
IB	Internal bond strength
TS	Thickness swelling
